# Supplementary material for: Prevalent genetic alterations in pediatric thyroid carcinoma: Insights from an Argentinean study
Source: PLoS One. 2025 May 8;20(5):e0323271. doi: 10.1371/journal.pone.0323271 (PMC12061146; doi:10.1371/journal.pone.0323271)
Supplement: S1 Table — Both sexes, per 100.000 for representative countries around the globe. (DOCX) [file pone.0323271.s001.docx]

| **S1 Table. Estimated Age-Standardized Incidence Rates (ASR) for thyroid cancer.** Both sexes, per 100.000 for representative countries around the globe. | | | | | |
| --- | --- | --- | --- | --- | --- |
| **Geographic Region** | **Country** | **Age groups (years)** | | | |
|  |  | **0 - 14** | **15 - 19** | **0 - 19** | **20 - 85+** |
| South America | Argentina | 0.05 | 1.7 | 0.43 | 13.1 |
|  | Brazil | 0.21 | 2.7 | 0.77 | 19.4 |
|  | Peru | 0.16 | 1.3 | 0.42 | 11.5 |
| North America | Mexico | 0.42 | 2.6 | 0.90 | 12.9 |
|  | United States of America | 0.36 | 3.2 | 1.00 | 19.0 |
| Europe | United Kingdom | 0.20 | 2.4 | 0.70 | 9.7 |
|  | Spain | 0.20 | 2.0 | 0.61 | 12.0 |
|  | France | 0.37 | 4.1 | 1.2 | 23.8 |
|  | Germany | 0.23 | 1.7 | 0.58 | 8.5 |
| Eastern Asia | Russian Federation | 0.15 | 1.8 | 0.53 | 11.3 |
|  | China | 0.13 | 2.4 | 0.64 | 18.5 |
|  | Japan | 0.09 | 1.4 | 0.37 | 13.2 |
|  | Thailand | 0.12 | 2.4 | 0.63 | 6.2 |
| Oceania | Australia | 0.15 | 2.6 | 0.71 | 18.5 |
| Western Asia | India | 0.05 | 0.54 | 0.16 | 2.3 |
|  | Islamic Republic of Iran | 0.13 | 1.5 | 0.43 | 7.0 |
|  | Turkey | 0.36 | 3.7 | 1.1 | 23.0 |
|  | Saudi Arabia | 0.14 | 2.5 | 0.68 | 12.1 |
| Africa | Egypt | 0.01 | 0.24 | 0.06 | 4.8 |
|  | Morocco | 0.08 | 0.77 | 0.24 | 9.4 |
|  | Democratic Republic of the Congo | 0.00 | 0.07 | 0.02 | 1.3 |
|  | South Africa | 0.03 | 0.18 | 0.07 | 3.7 |
| Data source: GLOBOCAN 2020 and IARC - WHO: http://gco.iarc.fr/today | | | | | |
