# Supplementary material for: Prevalent genetic alterations in pediatric thyroid carcinoma: Insights from an Argentinean study
Source: PLoS One. 2025 May 8;20(5):e0323271. doi: 10.1371/journal.pone.0323271 (PMC12061146; doi:10.1371/journal.pone.0323271)
Supplement: S2 Table — (DOCX) [file pone.0323271.s002.docx]

| **S2 Table. Molecular alteration in adult and pediatric PTC** | | | |
| --- | --- | --- | --- |
|  | **Pediatric PTC** | | **Adult PTC** |
| **Molecular alteration** | **Worldwide** | **Argentina** | **Worldwide** |
| *NTRK3* fusion | 0 - 18 % | 7 % | 1 - 5 % |
| *NTRK2* or *NTRK1* fusion | 2 - 4 % | 0% | 0.8 - 1% |
| *RET* fusion | 0 - 57 % | 10.5 % | 3 - 20% |
| *ALK* fusion | 0 - 6.5% | 8.8 % | 0 - 7% |
| *MET* fusion | 1% | 1.8 % | 0% |
| *BRAF* fusion | 0 - 19 % | 1.8 % | 0 - 2 % |
| BRAF V600E SNV | 0 - 63 % | 12.3 % | 27 - 83 % |
| Worldwide frequencies were obtained from (Yoo, S. K. et al. 2016, Paulson, V. A. et al. 2019, Yoo, S. K. et al. 2019, Pekova, B. et al. 2020, Rangel-Pozzo, A. et al. 2020, Guleria, P. et al. 2022 and Bulanova Pekova, B. et al. 2023) | | | |
