# Supplementary material for: Prevalent genetic alterations in pediatric thyroid carcinoma: Insights from an Argentinean study
Source: PLoS One. 2025 May 8;20(5):e0323271. doi: 10.1371/journal.pone.0323271 (PMC12061146; doi:10.1371/journal.pone.0323271)
Supplement: S3 Table — (DOCX) [file pone.0323271.s003.docx]

| **S3 Table. FISH probe description** | | |
| --- | --- | --- |
| **Targeted gene** | **Probe name** | **Manufacturer / Catalog number** |
| *BRAF* | LIVe Oncogenic Breakapart BRAF Probe | Lexel / OBA 7q34 |
| *ALK* | LIVe Oncogenic Breakapart ALK Probe | Lexel / OBA 2p23 |
| *RET* | LIVe Oncogenic Breakapart RET Probe | Lexel / OBA 10q11 |
| *MET* | LIVe Oncogenic Breakapart MET Probe | Lexel / OBA 7q31 |
| *NTRK1* | Custom NTRK1 Breakapart Probe - OGT | CytoCell / MPP 15370 |
| *NTRK2* | Custom NTRK2 Breakapart Probe - OGT | CytoCell / MPP 15380 |
| *NTRK3* | Custom NTRK3 Breakapart Probe - OGT | CytoCell / MPP 15390 |
| *ETV6* | LIVe Oncogenic Breakapart ETV6 Probe | Lexel / OBA 12p13 |
|  |  |  |
